# Trehalose-mediated reshaping of the rhizosphere microbiome drives tea root rot progression

**DOI:** 10.3389/fmicb.2026.1787317

**Published:** 2026-02-19

**Authors:** Qiang Zhu, Bowen Chen, Weiting Hu, Yingbo Huang, Shengyuan Wang, Mei Feng, Jie Zhao, Mingyi Yu, Mingzhu Li, Xuejiao Gong

**Affiliations:** 1 Tea Research Institute, Sichuan Academy of Agricultural Sciences, Chengdu, China; 2 School of Food and Pharmaceutical Engineering, Guizhou Institute of Technology, Guiyang, Guizhou, China

**Keywords:** biocontrol, rhizosphere microbial community, root exudates, tea root rot disease, trehalose

## Abstract

Tea (*Camellia sinensis* [L.] Kuntze) is one of the most economically important crops and as a traditional medicinal plant in the world. The long-term continuous cropping and inappropriate management have led to frequent outbreaks of soil-borne diseases such as root rot, which pose a serious threat to the sustainable development of the tea industry. However, the pathogenesis of tea root rot remains poorly understood. In this study, two novel pathogen fungi, *Paraconiothyrium cyclothyrioides* F8 and *Apiotrichum sporotrichoides* F17, were isolated and identified from diseased tea roots. Microbiome analysis revealed significant restructuring of the rhizosphere microbial community in diseased tea plants, with a significant reduction in the abundance of *Basidiomycota* and marked enrichment of pathogen such as *Fusarium* and *Apiotrichum*. Meanwhile, the abundances of beneficial fungi (e.g., *Saitozyma* and *Trichoderma*) and bacteria (e.g., *Bacillus* and *Sporosarcina*) were significantly decreased. Further investigation demonstrated that root exudate trehalose exhibited prominent bidirectional regulatory effect through promoted the growth of pathogen, while simultaneously inhibiting biofilm formation, rhizosphere colonization at specific concentrations and weakened the biocontrol functions of the beneficial antagonistic bacteria *Sporosarcina pasteurii* T21 and *Lysinibacillus* sp. T23, facilitating the formation of a rhizosphere chemical environment that “aids enemies and harms allies” and thereby exacerbating disease occurrence. This study emphasized the dominant role of plant metabolites such as trehalose in driving the assembly of rhizosphere microbial communities from a disease-suppressive to a disease-conducive state, as well as in disease development. The findings provide a novel theoretical perspective for the microbiological regulation of tea root rot and offer theoretical and practical bases for tea root rot disease green prevention and control.

## 1 Introduction

Tea (*Camellia sinensis* [L.] Kuntze) is an important economic crop in the world and one of the most widely consumed non-alcoholic beverages globally, with extensive cultivation in Asia, Africa, and South America (Bora and Bora, 2021). Additionally, tea plants are also as traditional medicinal plant has been widely used in traditional medicine and continues to hold significant value in modern health-promoting applications, due to tea leaves are rich in a variety of bioactive constituents including tea polyphenols, alkaloids, and flavonoids, which exhibit multiple health-related functions such as antioxidant, anti-inflammatory, and potential benefits in the prevention of cardiovascular diseases and tumors (Bortolini et al., 2021; Hong et al., 2022). However, long-term continuous cropping, frequent harvesting, and inappropriate field management have led to soil degradation and frequent outbreaks of soil-borne diseases in tea plants. Root rot disease is one of the most devastating and challenging to control, as severe outbreaks can result in substantial economic losses (Elango et al., 2015; Pandey et al., 2021). Tea root rot disease is caused by various pathogen, such as *Armillaria mellea*, *Candida* spp., and *Fomes lamoensis*, which leads to blackened and rotting roots in tea plants, followed by chlorosis and wilting of leaves, and even result in plant death (Mishra et al., 2014; Morang et al., 2018). In recent years, the increasing occurrence and severity of tea root rot disease, driven by global warming and the rising frequency of extreme weather events, have led to the incidence rate reaching up to 50% in some tea gardens, which poses a significant threat to the global tea industry (Hong et al., 2018). Currently, chemical fungicides remain the primary method for preventing and controlling tea root rot. However, their application has raised serious concerns regarding environmental pollution, pesticide residues, and the development of pathogen resistance, which may even compromise consumer health (Murugavel et al., 2025). Therefore, the development and application of safe, efficient, and sustainable environmentally friendly strategies against tea root rot have become an urgent requirement for the sustainable development of the tea industry.

As the core interface of plant–soil-environment interactions, the rhizosphere microbiome plays an irreplaceable role in promoting plant growth, enhancing nutrient absorption, and improving plant disease resistance through complex metabolic networks and ecological functions, and is referred to as the “second genome” of plants (Sun et al., 2023; Xun et al., 2024). A large number of studies have demonstrated that the stability and functional diversity of rhizosphere microbial community directly determine the plant health and disease resistance (Gao et al., 2021; Qu et al., 2020; Sun et al., 2024). For example, rhizosphere microbiome enriched with protective bacteria such as *Firmicutes* and *Actinomycetes* enhances the suppression of *Ralstonia solanacearum* and reduces the incidence of tomato wilt (Kwak et al., 2018). Similarly, rice plants inoculated with beneficial endophytic fungi have been shown to enrich protective *Pseudomonas* spp. to resist pathogen infection and reducing the severity of rice spikelet rot disease (Zhu et al., 2023). Compared to diseased plants, the rhizosphere of healthy plants typically exhibits higher microbial community richness and diversity, while also enriching more beneficial microbial groups such as *Pseudomonas*, *Bacillus*, *Streptomyces*, and *Trichoderma* (Trivedi et al., 2022). These microorganisms assist plants in resisting pathogen invasion through competing for ecological niches, secreting antibacterial substances or inducing plant resistance (Spooren et al., 2024; Toju et al., 2018). The role of tea plant microbiota in regulating tea quality, defending against pathogens, and promoting plant growth has been documented in previous studies (Chen et al., 2023; Tang et al., 2025). However, the contribution of the rhizosphere microbiome to plant health, as well as how its microbial community responds to pathogen challenge remains unclear.

In fact, the assembly of rhizosphere microbial communities is largely shaped by the host plant secreting various rhizosphere metabolites, including root exudates and microbial metabolites, as signals and nutrient to regulate the assembly of the rhizosphere microbiome, and attract specific microbial groups to adapt to the environment and maximize resource utilization (Koprivova and Kopriva, 2022; Yue et al., 2023). For instance, transient disruption of the Casparian strip in root endodermal barriers can lead to glutamate leakage onto the root surface, recruiting specific soil bacteria for colonization (Tsai et al., 2025). The aerial roots of sorghum plants also secrete carbohydrate-rich mucilage into the soil to recruit nitrogen-fixing bacteria, enabling the plants to obtain 40% of their nitrogen from the atmosphere (Venado et al., 2025). Similarly, flavonoids secreted by legume plants strongly influence the *Arabidopsis thaliana* rhizosphere microbiome by attracting *Aeromonas* spp., to enhance plant resistance to dehydration stress (He et al., 2022). As a plant rich in various natural metabolites, the tea plant may shape a microbial environment more beneficial to its health through unique secondary metabolites (Jibola-Shittu et al., 2024). Recent studies have shown that L-theanine secreted by tea roots modulates soil element cycling by shaping the rhizosphere microbiome, significantly inhibiting the soil denitrification and complete nitrification pathways (Xie et al., 2022). The specific metabolites such as theophylline (TP) and epigallocatechin gallate (EGCG), produced during tea bud, have also been proven to drive the assembly of the epiphytic microbial community and continuously inhibit above-ground pathogens (Xu et al., 2022). However, there are few studies on how root exudates influence plant resistance to root rot disease by regulating the structure and function of the tea plant rhizosphere microbiome.

Therefore, this study systematically compared the differences in root exudates between healthy and root rot-infected tea plants, as well as their effects on rhizosphere bacterial and fungal communities. Furthermore, we identified the key metabolites driving microbial community assembly and pathogen infection, and verified their functions in disease resistance through exogenous addition experiments. This research aims to elucidate the underlying relationship and mechanisms of the “root exudates–rhizosphere microbiome–plant disease resistance” in tea plants during defense against root rot, which can provide theoretical basis and experimental foundation for developing green control strategies for tea root rot disease based on the targeted optimization of the root microenvironment.

## 2 Materials and methods

### 2.1 Sample collection

Samples were collected in May 2024 (spring tea season) from a tea garden (30°N,103°E) affected by root rot in Ya’an City, Sichuan Province, China. The plantation had been cultivated with the tea cultivar ‘Fuxuan 9’ for an extended period. Experiments were conducted with two treatments: (a) rhizosphere soil of healthy plant (NF), (b) rhizosphere soil of diseased plant (CF). Tea plant individuals infected with root rot were randomly selected, after removing approximately 5 cm of surface soil, the root systems were carefully excavated, then non-rhizosphere soil was shaken off, and the soil tightly adhering to the roots (≤2 mm) was collected as rhizosphere soil and placed into sterile 50 mL centrifuge tubes. Meanwhile, rhizosphere soil from healthy tea plants was also collected randomly. All samples were replicated three times and stored at 4 °C, and some samples were frozen at −80 °C for future use.

### 2.2 Isolation and identification of pathogen

Pathogens were isolated from tea plant roots infected with root rot using the tissue isolation method. Briefly, a 2 cm segment of diseased tea roots were cut with sterile scissors, surface-sterilized by 75% ethanol for 30 s and 0.1% NaClO for 30 s, and then rinsed five times with sterile water. The disinfected root segments were inoculated on potato dextrose agar (PDA) medium (200 g/L potato, 20 g/L glucose, 15 g/L agar, pH 7.0) and cultured in the dark at 28 °C for 5 days. The pathogens were identified based on morphological characteristics, conidial features, and sequence analysis of the nuclear ribosomal ITS1 (5’-TCCGTAGGTGAACCTGCGG-3′) and ITS4 (5’-TCCTCCGCTTATTGATATGC-3′). The PCR products were sequenced by Sangon Biotech (Shanghai, China) Co., Ltd., and the sequences were subjected to BLAST alignment in GenBank.

For the isolated potential pathogenic fungi, artificial inoculation was performed through pot experiments to assess their ability to induce tea root rot symptoms. Additionally, Koch’s postulates were strictly followed for pathogenicity determination, including re-isolating fungi from diseased tissues to confirm the pathogens.

### 2.3 Soil microbiome analysis

Total DNA was extracted from 0.5 g of rhizosphere soil collected from both diseased and healthy tea plants using the FastDNA™ Spin Kit for Soil (MP Biomedicals, CA, USA). The V4 region of the bacterial 16S rRNA gene was amplified with primers 515F (5’-GTGCCAGCMGCCGCGGTAA-3′)/805R (5’-GGACTACHVGGGTWTCTAAT-3′), and the fungal ITS1 region was amplified with primers ITS1F (5’–CTTGGTCATTTAGAGGAAGTAA-3′)/ITS2R (5’GCTGCGTTCTTCATCGATGC-3′). Amplicon sequencing was performed on the Illumina NovaSeq 6000 Sequencer (Illumina, Inc., CA). Quality control, denoising, and operational taxonomic unit (OTU) table generation were conducted using the QIIME 2 (1.91) and DADA2 pipelines. Taxonomic annotation was conducted based on the SILVA 138 database (for bacteria) and the UNITE 8 database (for fungi), *α*-diversity and *β*-diversity were calculated, LEfSe was used to identify microbial groups with significant differences between groups.

### 2.4 Isolation and identification of rhizosphere microorganisms

10 g rhizosphere soil collected from diseased tea plants was mixed with 90 mL of sterile 0.1 M phosphate-buffered (PBS) and shaken at 180 rpm for 30 min. 100 μL of the suspension was diluted to appropriate concentrations and spread onto different bacterial culture media (LB, TSA, and NB) using sterile spreader, the plates were incubated at 37 °C for 48 h. Single colonies with different morphological characteristics were selected for purification culture and stored in 20% glycerol tubes at −80 °C. The bacterial 16S rRNA gene was amplified using the primers 27F (5’-AGAGTTTGATCTGGCTCAG-3′) and 1492R (5’-GGTTACCTTGTTACGACTT-3′), then perform BLAST sequence alignment in GenBank.

### 2.5 Antagonistic activity of isolated microorganisms

The inhibitory effect of rhizosphere microorganisms on pathogen was evaluated using the plate confrontation method. A 5 mm diameter fungal disks was placed on one side of a PDA plate, while the test bacteria were inoculated on the opposite side. Plates were incubated at 28 °C for 5 days, and the diameter of the pathogen colony was measured. Plates inoculated with pathogen alone was used as the control, and all assays were performed in triplicate. Calculate the inhibition rate of colony diameter:


$$\begin{array}{l} \text{Inhibition rate}=(\begin{array}{l} \text{Control colony diameter}-\\ \text{Treatment colony diameter} \end{array})/\\ \text{Control colony diameter}\times 100\% \end{array}$$

### 2.6 Antagonistic microorganism disease control experiment

To evaluate the control effect of antagonistic microorganisms against tea root rot, a pot experiment was conducted under controlled conditions. Tea seedlings with consistent height (13 ± 2 cm) and no pest and disease damage were selected for the experiment, and the tea seedlings were randomly divided into different treatment groups to ensure the homogeneity of the experimental materials. To simulate the complex infection of root rot by multiple pathogens in natural environments, a combined infection group consisting of two pathogen (named as SY) was established. Meanwhile, a simple synthetic microbial community composed of two antagonistic bacterial strains (named as SC) was constructed. For each pathogen treatment, four experimental groups were set up: (1) CK (only inoculated with pathogen); (2) T21 (inoculated with pathogen+ antagonistic bacteria T21); (3) T23 (inoculated with pathogen+ antagonistic bacteria T23); (4) SC (inoculated with pathogen+ T21 + T23). The experiment included three pathogen treatments, resulting in 12 treatments were set up. At least three independent experiments were conducted, each treatment was replicated three times, and each pot was planted with three seedlings (tea cultivar “Fuxuan 9”) grown in sterilized soil, so totaling at least 27 plants per treatment.

Plant roots were first treated with 2 mL of bacterial suspension (10^8^ CFU mL^−1^), after 3 days, 2 mL of pathogen spore suspension (10^7^ CFU mL^−1^) was drenched into the rhizosphere soil surrounding the roots. The incidence rate = Number of plants showing typical root rot symptoms/Total number of inoculated plants × 100%. Disease severity was assessed 30 days after pathogen inoculation (grading standard: 0 grade without disease, 1 grade root discoloration <20%, 2 grade 20–50%, 3 grade >50%, 4 grade complete root rot), and the plant height, root length and fresh weight of the tea seedlings were measured.

### 2.7 Plant defense indicator determination

#### 2.7.1 Defense enzyme activity

0.5 g fresh root sample was placed in a pre-chilled mortar with 5 mL of ice-cold 50 mM potassium phosphate buffer (PBS, pH 7.0), and grind into a homogenate under ice-bath conditions. The homogenate was transferred to a 10 mL centrifuge tube and centrifuged at 12,000 r·min^−1^ for 20 min at 4 °C. The activities of POD enzymes were determined using commercial kits (Nanjing Jiancheng Bioengineering Institute, Nanjing, China). Each sample was amplified in triplicate in each experiment.

#### 2.7.2 JA content assay

1.0 g of fresh tea plant root sample was ground in liquid nitrogen, and extracted with 5 mL pre-chilled methanol by vortexing for 30 s, followed by dark incubation at 4 °C for 12 h. Then centrifuged at 8,000 r·min^−1^ at 4 °C for 15 min, and the supernatant was collected. The residue was re-extracted with 3 mL of cold methanol, and the combined supernatants were evaporated at 45 °C until methanol was completely removed. JA content was determined following the method described by Zhang et al. (2019).

### 2.8 Soil metabolome analysis

0.5 g rhizosphere soil samples from diseased and healthy tea plants were extracted with 1 mL of ice-cold extraction solvent (methanol: water: acetonitrile = 2:2:1, v/v/v) containing an internal standard mixture (including L-2-chlorophenylalanine). Then vortex oscillation for 30 s and subjected to ultrasonic extraction in an ice-water bath for 30 min. The mixture was standing at −20 °C for 1 h, then centrifuged at 13,000 rpm for 15 min at 4 °C. The supernatant was filtered through a 0.22 μm microfilters and stored at −80 °C, then sent to Majorbio Bio-pharm Technology (Shanghai, China) Co., Ltd. for analysis.

### 2.9 Effect of key compounds on antagonistic microorganisms

#### 2.9.1 Effect of key compounds on the growth of antagonistic microorganisms

Correlation analysis was performed between the microbiome and metabolome results, and finally six key differential compounds (D-galactose, trehalose, uridine, galactinol, xylobiose, and panose) that were significantly correlated with pathogens and bacteria were selected for further analysis. Each compound was prepared as a 1 g/L stock solution and sterilized by 0.22 μm filter membrane for subsequent testing.

Antagonistic microorganisms were inoculated into liquid minimal medium (MM) containing key compounds at final concentrations of 0, 1, 10, and 100 mg/L (medium composition: glucose 1 g, K₂HPO_4_ 7 g, KH_2_PO_4_ 3 g, (NH_4_)_2_SO_4_ 3 g, MgSO_4_·7H_2_O 100 mg, agar 15 g; pH 7.0 ± 0.2). The initial OD_600_ of each culture was 0.1, and the cultures were cultured at 37 °C, 180 r/min. The OD_600_ values were measured at 4 h and 12 h of bacterial growth. The concentrations of the compounds were set according to literature reports, and each experiment was repeated three times.

#### 2.9.2 Effect of key compounds on biofilm production of antagonistic microorganisms

The determination of biofilms was carried out using crystal violet staining method. Briefly, 20 μL of a bacterial culture diluted to an OD_600_ = 0.01 was added to added to 180 μL of minimal medium (MM) containing key compounds at final concentrations of 0, 1, 10, and 100 mg/L in a 96-well cell culture plate, with a final volume of 200 μL per well. The plate was sealed and cultured at 37 °C for 48 h. The supernatant was discarded, and the wells were carefully rinsed three times with PBS, 150 μL of crystal violet staining solution was added for staining 15 min, then decolorization was performed with 33% glacial acetic acid, and the OD₅₉₀ value was measured (Kim et al., 2021). Each experiment was repeated three times.

#### 2.9.3 Effect of key compounds on motility of antagonistic microorganisms

For the motility assay, 2 μL of bacterial culture diluted to OD_600_ = 0.01 was inoculated at the center of 0.6% MM agar plates containing key compounds at final concentrations of 0, 1, 10, and 100 mg/L. After incubation at 37 °C for 24 h, the diameter of bacterial growth was measured. Each experiment was repeated three times.

### 2.10 Effect of key compounds on pathogen growth

A 5 mm diameter pathogen disks was inoculated at the center of Czapek’s medium (NaNO_3_, 3 g; K_2_HPO_4_, 1 g; MgSO_4_·7H_2_O, 0.5 g; KCl, 0.5 g; FeSO_4_·7H_2_O, 0.01 g; sucrose, 30 g; Agar, 15 g, pH 7.0) containing key compounds at final concentrations of 0, 1, 10, and 100 mg/L. The plates were incubated at 28 °C for 5 days and the colony diameter was measured. Each experiment was repeated three times.

### 2.11 Disease resistance experiment of key compounds combined with antagonistic microorganisms

To assess the influence of key compounds on the efficacy of antagonistic microorganisms in controlling root rot, a pot experiment was conducted under controlled conditions. This experiment focused specifically on combined pathogen infection. Eight treatment groups were set up: (1) SY (inoculated with F8 + F17); (2) T21 (inoculated with SY + antagonistic bacterium T21); (3) T23 (inoculated with SY + antagonistic bacterium T23); (4) SC (inoculated with SY + antagonistic bacteria T21 + T23); (5) Cp (inoculated with SY + Trehalose); (6) Cp21 (inoculated with SY + Trehalose+ T21); (7) Cp23 (inoculated with SY + Trehalose+ T23); (8) CpSC (inoculated with SY + Trehalose+ T21 + T23). Each treatment was replicated three times., and each pot was planted with three seedlings (tea cultivar “Fuxuan 9”) grown in sterilized soil. The plant treatment was conducted according to the above experiment.

### 2.12 Statistical analysis

All statistical analyses were performed using SPSS 20.0 (SPSS Inc., Chicago, IL, USA). Data are presented as mean ± standard error (SE). Multiple comparisons were conducted by one-way analysis of variance (ANOVA), followed by the least significant difference (LSD) interval test to compare data and show differences between means, and Tukey’s HSD test was used to determine significance levels (*p < 0.05, **p < 0.01, ***p < 0.001). Other statistical analyses and graphs were generated using GraphPad 8.0 (GraphPad Software, Boston, Massachusetts, USA).

## 3 Results

### 3.1 Multiple pathogen infections cause tea root rot disease

**Figure 1:**
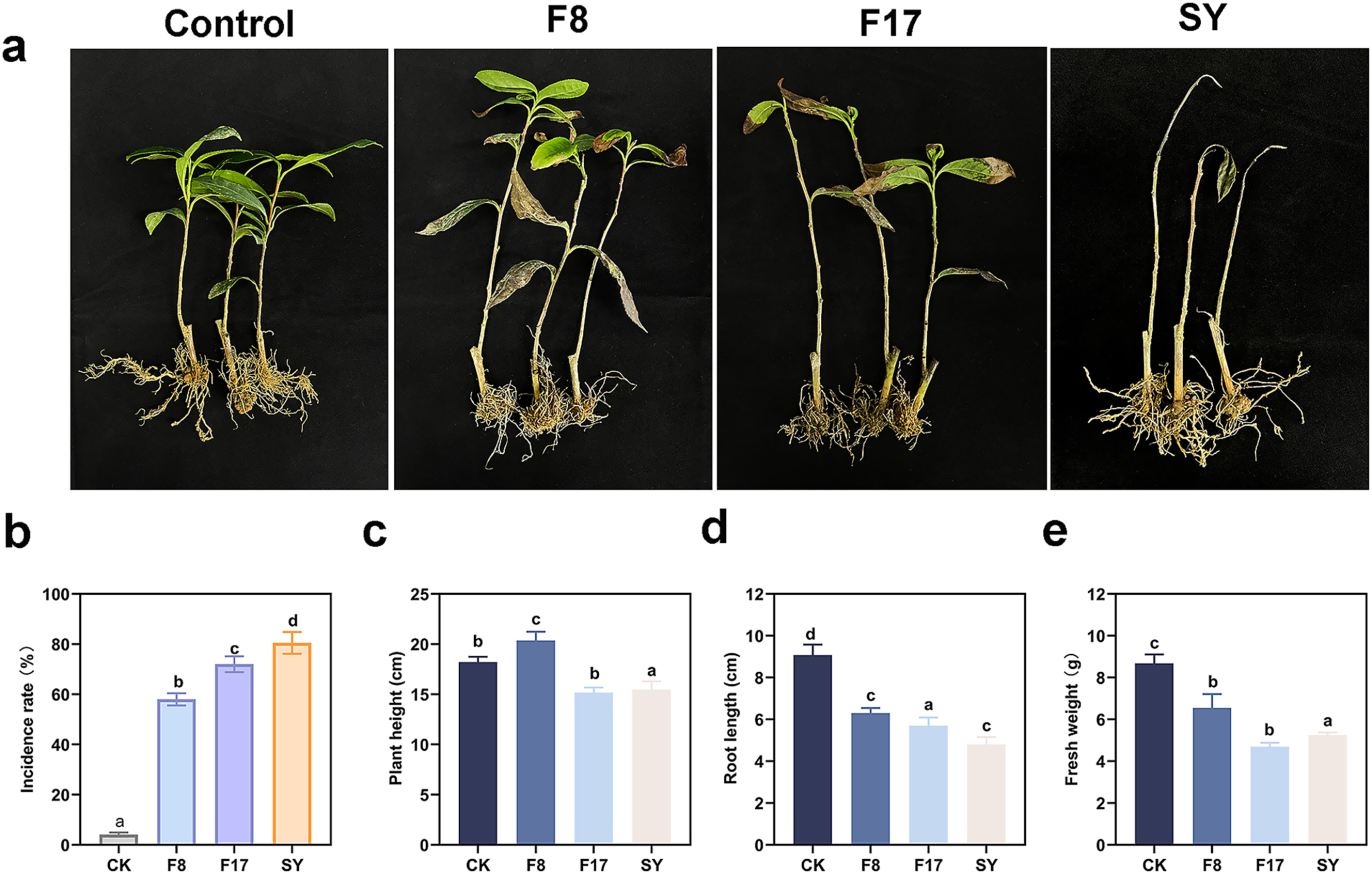
Infection symptoms of the different pathogen causing tea root rot disease. **(a)** Photos of infections caused by different pathogens (F8, F17, and SY). **(b)** The incidence rate of root rot caused by different pathogen. **(c)** The plant height. **(d)** Root length. **(e)** Fresh weight of tea infected by different pathogen. SY refers to a combined infection group consisting of two pathogens (F8 and F17).

To determine the pathogen causing tea root rot disease, we isolated and identified the pathogen from the roots of the diseased plant (Figure 1). Two pathogen strains that could induce root rot symptoms were successfully isolated, and they were identified as *Paraconiothyrium cyclothyrioides* (F8) and *Apiotrichum sporotrichoides* (F17). Although the disease symptoms induced by the two pathogens were not identical, both blackening or browning of tea seedling roots, accompanied by leaf yellowing and abscission (Figure 1a). Compared with healthy plants, the incidence of root rot caused by F8 was 58%, that caused by F17 was 72%, and up to 80.5% under multi-infection (Figure 1b). Although F8 infection promoted above-ground growth of plant, both pathogens significantly reduced plant height and fresh weight compared to the healthy control (Figures 1c–e).

### 3.2 Pathogen infection reshapes the rhizosphere microbiome

**Figure 2:**
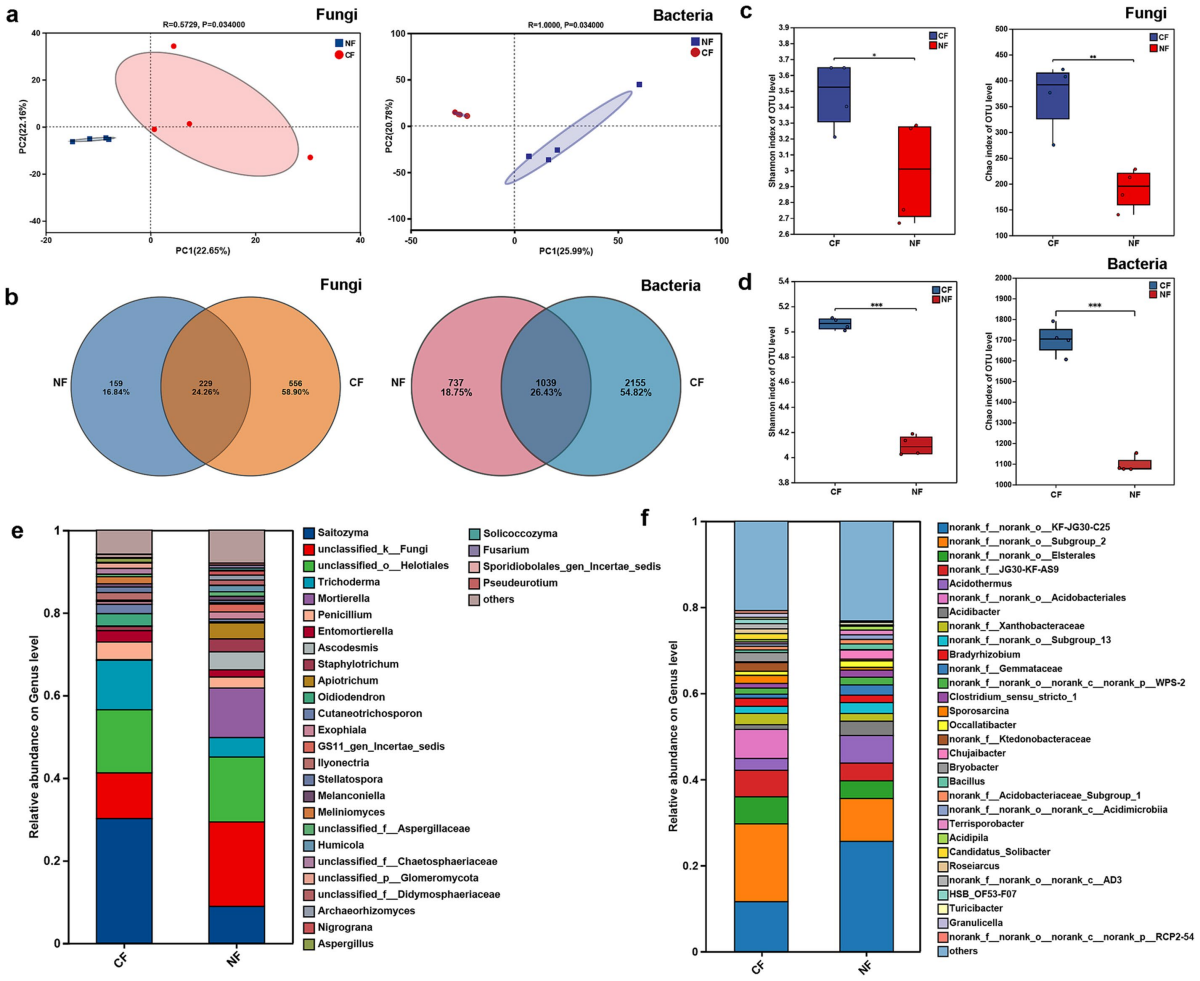
Characteristics of microbial communities in the rhizosphere of healthy and diseased tea plants. **(a)** Principal component analysis (PCA) of fungi and bacteria. **(b)** Venn diagram analysis of fungi and bacteria. **(c)** *α*-Diversity analysis of fungi. **(d)** α-Diversity analysis of bacteria. **(e)** Composition of the fungal rhizosphere microbiome in tea plant. **(f)** Composition of the bacteria rhizosphere microbiome in tea plant.

To clarify the differential characteristics of rhizosphere microbial communities, we analyzed the composition and structure of microbial communities in the rhizosphere of diseased and healthy plants. As shown in Figure 2a, the principal component analysis (PCA) results indicated that the microbial communities of both bacterial and fungal communities in the diseased plants and healthy plants showing significant inter-group separation and clustered into independent branches, suggesting that pathogen infection significantly altered the structure of the rhizosphere microbial community. The *α*-diversity analysis further revealed the changes in community richness, both the Shannon and Chao index indicated that the rhizosphere bacterial and fungal communities of diseased plants had significantly higher richness than healthy plants (Figures 2c,d). Venn diagram analysis also supported this result that the rhizosphere of diseased plants contained more unique microbial taxa (Figure 2b).

Analysis of the rhizosphere fungal community composition revealed that *Ascomycota*, *Basidiomycota*, and *Mortierellomycota* were the dominant phyla in the tea rhizosphere, while the abundance of *Basidiomycota* was significantly lower in the rhizosphere of diseased plants (Figure 2e). At the genus level, the abundances of *Ascodesmis*, *Apiotrichum*, *Staphylotrichum*, *Nigrograna* and *Fusarium* significantly increased in diseased plants, whereas the abundances of *Saitozyma*, *Meliniomyces*, and *Aspergillus* were notably decreased. In contrast, the rhizosphere fungal communities of healthy plants were dominated by *Saitozyma*, *Trichoderma*, *Penicillium* and *Entomortierella* (Supplementary Figure S2). Analysis of the rhizosphere bacterial community indicated Proteobacteria, Acidobacteriota and Chloroflexi were the dominant bacterial phyla. The abundances of *Acidobacteriota*, *Verrucomicrobiota*, *Gemmatimonadota* and *Myxococcota* were significantly higher in the rhizosphere of diseased plants, while healthy plants exhibited a higher relative abundance of *Proteobacteria* (Figure 2f). At the genus level, the abundances of *Acidothermus*, *Acidibacter*, *Chujaibacter* and *Bacillus* were enriched in healthy plants (Supplementary Figure S2). These results indicate that pathogen infection markedly alters the composition, structure and diversity of the tea rhizosphere microbiome, which may further influence plant resistance and the development of root rot disease.

### 3.3 Antagonizing microorganisms to control tea root rot disease

**Figure 3:**
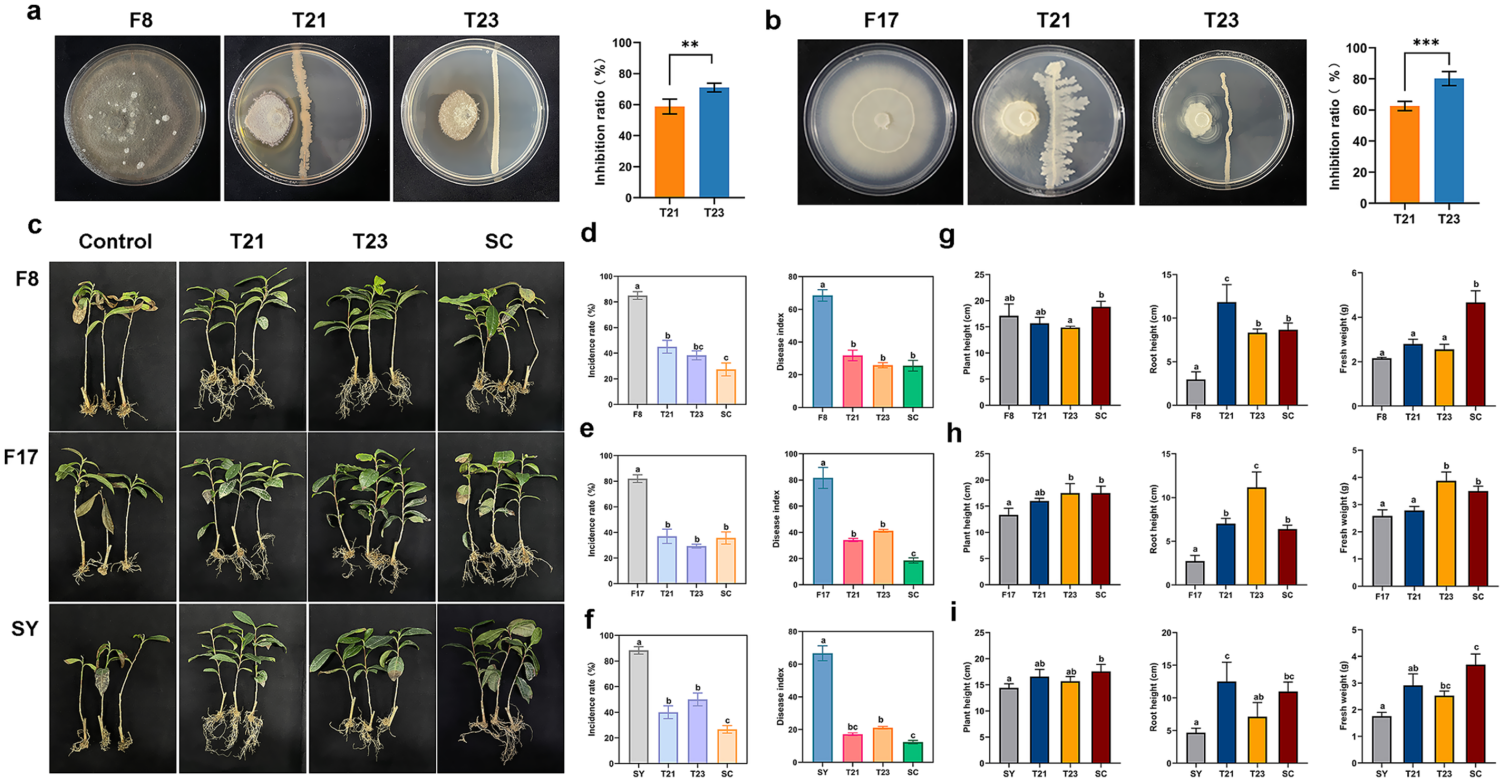
The control effect of antagonistic microorganisms on tea root rot disease. **(a,b)** The antagonistic activities in dual culture assays of T21 and T23 to F8 and F17. **(c)** Photos of the antagonistic microorganism control tea root rot disease. **(d)** The incidence rate **(g)** plant growth of antagonistic microorganisms on root rot disease by F8. **(e)** The incidence rate **(h)** plant growth of antagonistic microorganisms on root rot disease by F17. **(f)** The incidence rate **(i)** plant growth of antagonistic microorganisms on root rot disease by SY. SY refers to a combined infection group consisting of two pathogens (F8 and F17). SC refers to a simple synthetic microbial community composed of two antagonistic bacterial strains.

To explore the potential contribution of rhizosphere microorganisms to control tea root rot, 56 culturable bacterial strains were isolated from the health plants. These isolates antagonistic activity against pathogenic strains was evaluated *in vitro*, and leading to the identification of two strains, named as T21 (*Sporosarcina pasteurii*) and T23 (*Lysinibacillus* sp.), with pronounced inhibitory effects (Figure 3). As shown in Figure 3a, dual-culture plate demonstrated that both T21 and T23 exhibited significant antagonistic activity against the pathogens F8 (*Paraconiothyrium cyclothyrioides*) and F17 (*Apiotrichum sporotrichoides*). Specifically, the inhibition rates of T21 against F8 and F17 were 58.90 and 61.45%, respectively, while T23 showed inhibition rates of 72.46% against F8 and 80.32% against F17 (Figures 3a,b).

To further evaluate the disease-control potential of the antagonistic bacteria, a pot inoculation experiment was conducted under greenhouse conditions (Figure 3c). The results showed that, regardless of whether it was a single pathogen infection or a combined infection, inoculating the antagonistic bacteria T21 or T23 could significantly reduce the incidence and disease index of root rot disease. The results showed that both T21 and T23 significantly reduced the root rot whether the plants were subjected to single or multi-infection. Notably, the synthetic microbial community consisting of T21 and T23 exhibited superior control efficacy. Especially in multi-infection, the incidence and disease index in plants inoculated with the synthetic microbial community were only 26.67 and 12.33%, respectively, while in pathogen-only plants reached 88.33 and 66.67% (Figures 3d–i). Moreover, the antagonistic bacteria effectively alleviated the disease symptoms such as root rot and growth stagnation, and significantly promoting the development of tea tree roots and plant growth. Furthermore, individual inoculation with T21 or T23 slightly but significantly elevated the activity of defense-related enzymes and the content of jasmonic acid (JA), with T23 generally eliciting a stronger defense response than T21, which was consistent with previously result (Supplementary Figure S3). In all infection settings, the consortium consistently outperformed either single strain. Collectively, these findings highlight the critical role of combined inoculation with multiple antagonistic strains over single-strain inoculation in root rot management, whether under single-infection or multi-infection. However, the assembly mechanisms and regulatory factors governing the rhizosphere microbial community remain to be further elucidated.

### 3.4 Pathogen alters the tea root exudates

**Figure 4:**
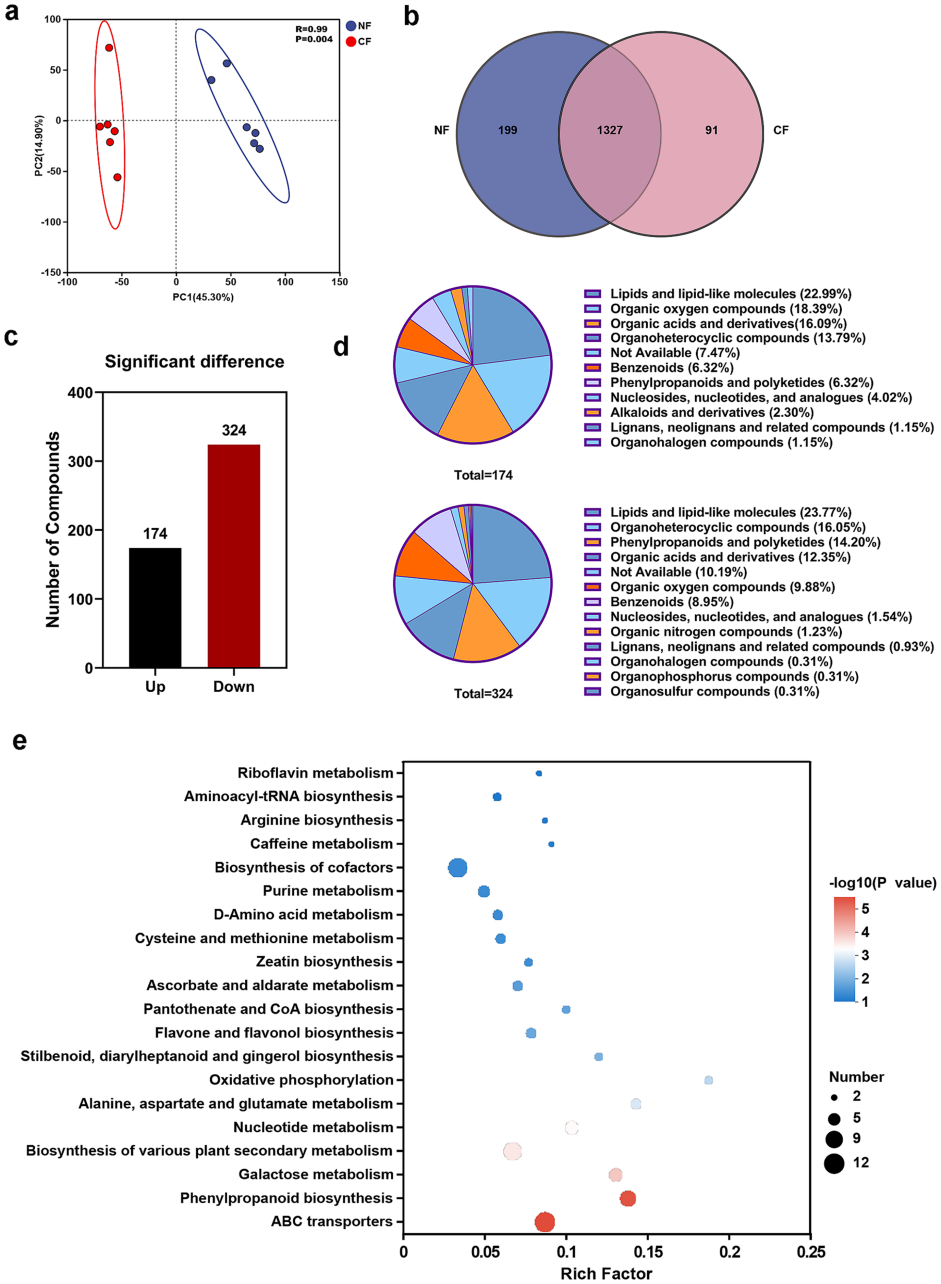
Characteristics of metabolite composition in the rhizosphere of healthy and diseased tea plants. **(a)** Principal component analysis (PCA). **(b)** Venn diagram analysis. **(c)** The number of differential metabolites. **(d)** The types and percentages of differential metabolites with increase (174) and decrease (324). **(e)** KEGG pathway analysis of differential metabolites.

The influence of plant metabolites on the assembly of rhizosphere microbial communities has been widely recognized. To explain the potential metabolic driving mechanisms underlying the differentiation of rhizosphere microbial communities, we performed non-targeted metabolomics analysis of the rhizosphere soil from healthy and diseased plants (Figure 4). PCA revealed clear intra-group clustering and inter-group separation between the healthy and diseased samples, indicating significant differences in their rhizosphere metabolic profiles (Figure 4a). A total of 1,327 metabolites were identified as common components in the healthy and diseased tea, with 199 metabolites uniquely detected in the healthy tea, and 91 metabolites uniquely detected in the diseased tea (Figure 4b). Compared with healthy plants, 498 metabolites exhibited significant alterations (VIP > 1, *p* < 0.05) in the rhizosphere of diseased plants, among which 174 metabolites were significantly down-regulated and 324 were significantly up-regulated (Figure 4c). Based on metabolite classification and annotation, the 174 down-regulated metabolites were primarily categorized into Lipids and lipid-like molecules (22.99%), Organic oxygen compounds (18.39%), Organic acids and derivatives (16.09%), Organoheterocyclic compounds (13.79%), Benzenoids (6.32%), and Phenylpropanoids and polyketides (6.32%). In contrast, the 324 up-regulated metabolites mainly belonged to Lipids and lipid-like molecules (23.77%), Organoheterocyclic compounds (16.05%), Phenylpropanoids and polyketides (14.20%), Organic acids and derivatives (12.35%), and Organic oxygen compounds (9.88%) (Figure 4d).

Heatmap analysis visually illustrated the overall metabolic differences between the two groups, with the color gradient reflecting changes in relative abundance, darker blue indicating higher expression levels of differential metabolites. Specifically, the contents of D-Galactose, Uridine, Xylobiose, Panose, Glucomannan, Galactinol, and Amylopectin were significantly higher in the rhizosphere of diseased plants, whereas Piperdial, Dodemorph, Acotiamide, PGDM, and Ceranapril were more abundant in healthy plant rhizosphere (Supplementary Figure S4). Additionally, the rhizosphere of healthy plants also had elevated levels of stress resistance and growth regulation-related metabolites such as abscisic acid, gibberellins, and quercetin, even though these substances were not among the top 35 differential metabolites. KEGG pathway enrichment analysis revealed that the differential metabolites were significantly enriched in pathways including ABC transporters, Phenylpropanoid biosynthesis, and Galactose metabolism, suggesting that pathogen infection may disrupt the normal metabolic network of tea plants, thereby influencing the assembly of the rhizosphere microbial community (Figure 4e).

### 3.5 Root exudates are correlated with the rhizosphere microbial community of tea plants

**Figure 5:**
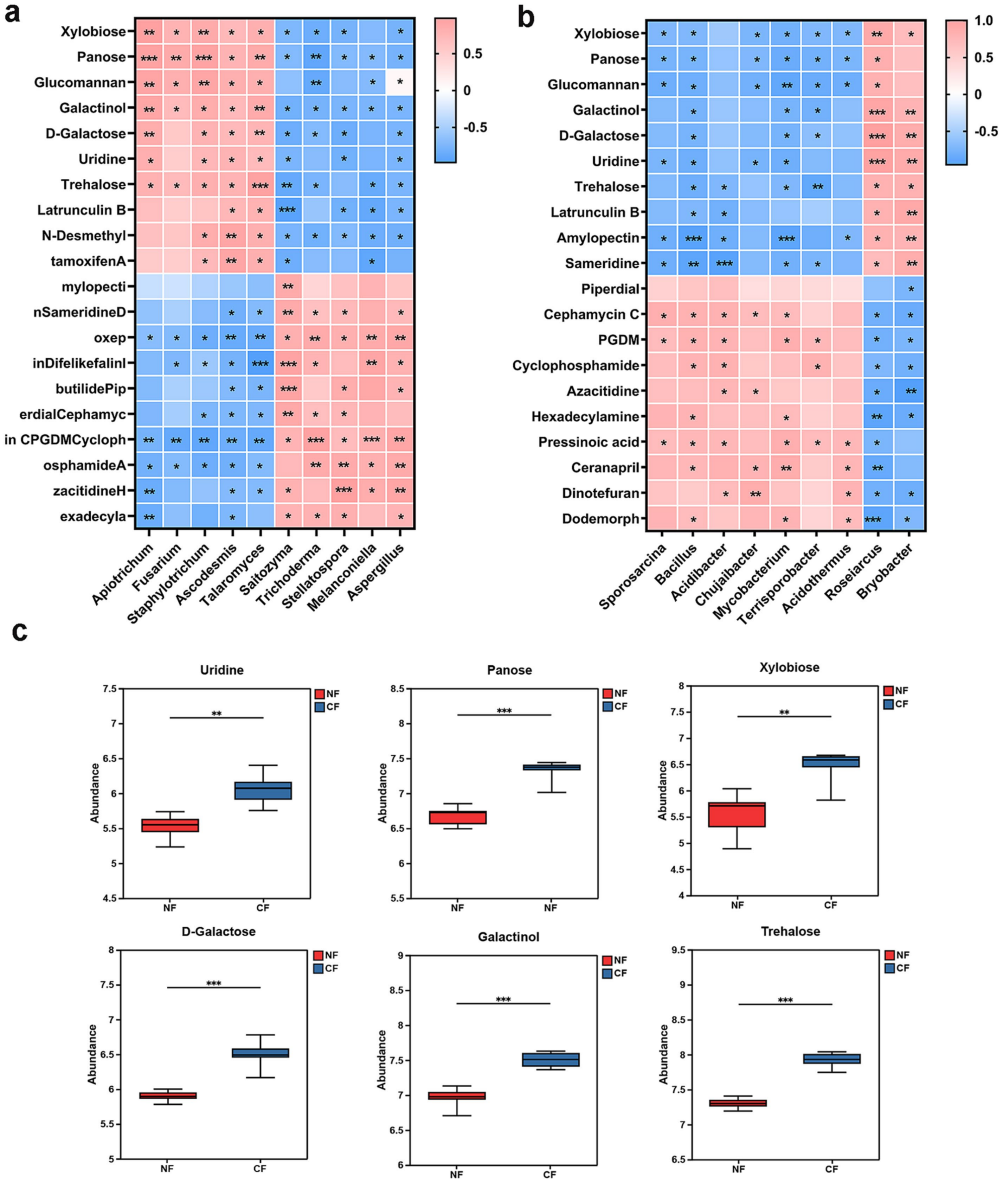
Correlation analysis of microbiomics and metabolomics. Heatmap of association analysis between the rhizosphere microbiome and differential metabolites **(a)** fungi **(b)** bacteria. **(c)** The relative content of the key compounds. The color scale represents correlation coefficients between microbial taxa and metabolites. Color intensity is proportional to the strength of association (red indicates positive correlation, blue indicates negative correlation).

Correlation analysis was performed between metabolite abundance and microbial community (Figure 5). The results revealed that the fungal genera *Apiotrichum*, *Fusarium*, *Staphylotrichum*, *Ascodesmis*, and *Talaromyces* showed significant positive correlations with compounds such as xylobiose, panose, glucomannan, galactinol, uridine, and trehalose, whereas they were significantly negatively correlated with *Saitozyma*, *Trichoderma*, *Stellatospora*, *Melanconiella*, and *Aspergillus* (Figure 5a). When focusing on rhizosphere bacteria, we found that xylobiose, panose, glucomannan, and uridine exhibited significant negative correlations with beneficial bacterial genera including *Sporosarcina*, *Bacillus*, and *Chujaibacter*. Trehalose displayed significant negative correlations with genera such as *Acidibacter*, *Bacillus*, *Mycobacterium*, and *Terrisporobacter*, while showing significant positive correlations with *Roseiarcus* and *Bryobacter* (Figure 5b). These findings suggest that these metabolites may promote the colonization and proliferation of pathogen while selectively modulating the growth of different functional groups of bacteria.

Based on the significance of correlations and microbial utilizability, six key metabolites (uridine, panose, xylobiose, D-galactose, galactinol, and trehalose) were selected for further investigation (Figure 5c). All these six compounds were significantly more abundant in the rhizosphere of diseased tea plants compared to healthy plants, indicating that they may play important roles in restructuring the rhizosphere microenvironment following pathogen infection.

### 3.6 Effect of key compounds on the antagonizing microorganisms

We further evaluated the effects of key metabolites on the growth and physiological characteristics of the antagonistic strains T21 and T23.

**Figure 6:**
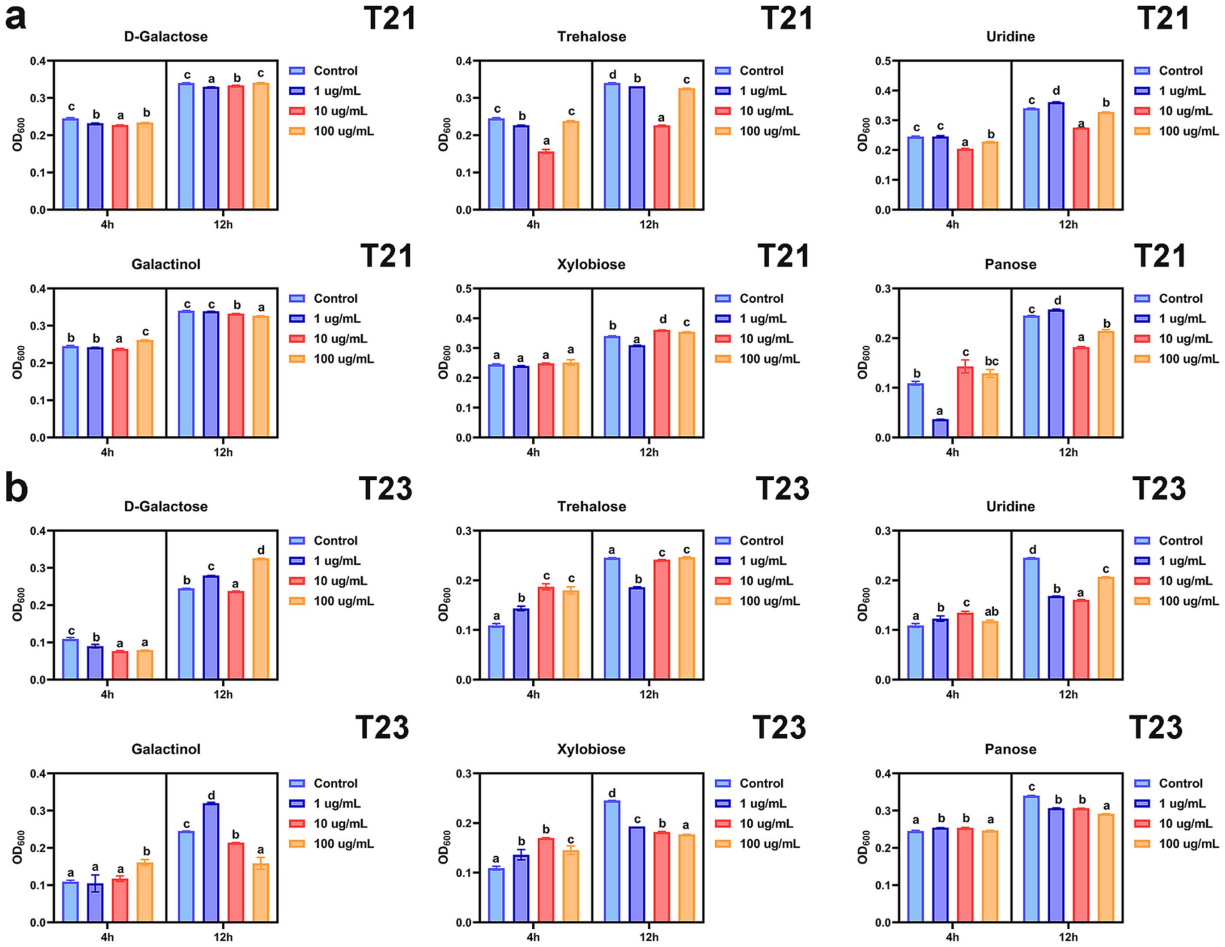
Effect of key compounds on the growth of antagonistic microorganisms: **(a)** T21 and **(b)** T23.

First, we determined the effects of metabolites at different concentrations on the growth of T21 and T23 (Figure 6). The results showed that all tested concentrations of D-galactose, trehalose, and uridine significantly inhibited the growth of T21 at both 4 h and 12 h (Figure 6a). In contrast, 100 μg/mL galactinol and 10 μg/mL panose promoted T21 growth at 4 h, while 10 μg/mL and 100 μg/mL xylobiose and 1 μg/mL panose enhanced T21 growth at 12 h, respectively (Figure 6b). As for T23, most compounds significantly inhibited its growth by 12 h, except for 100 μg/mL D-galactose and 1 μg/mL galactinol (Figure 6b).

**Figure 7:**
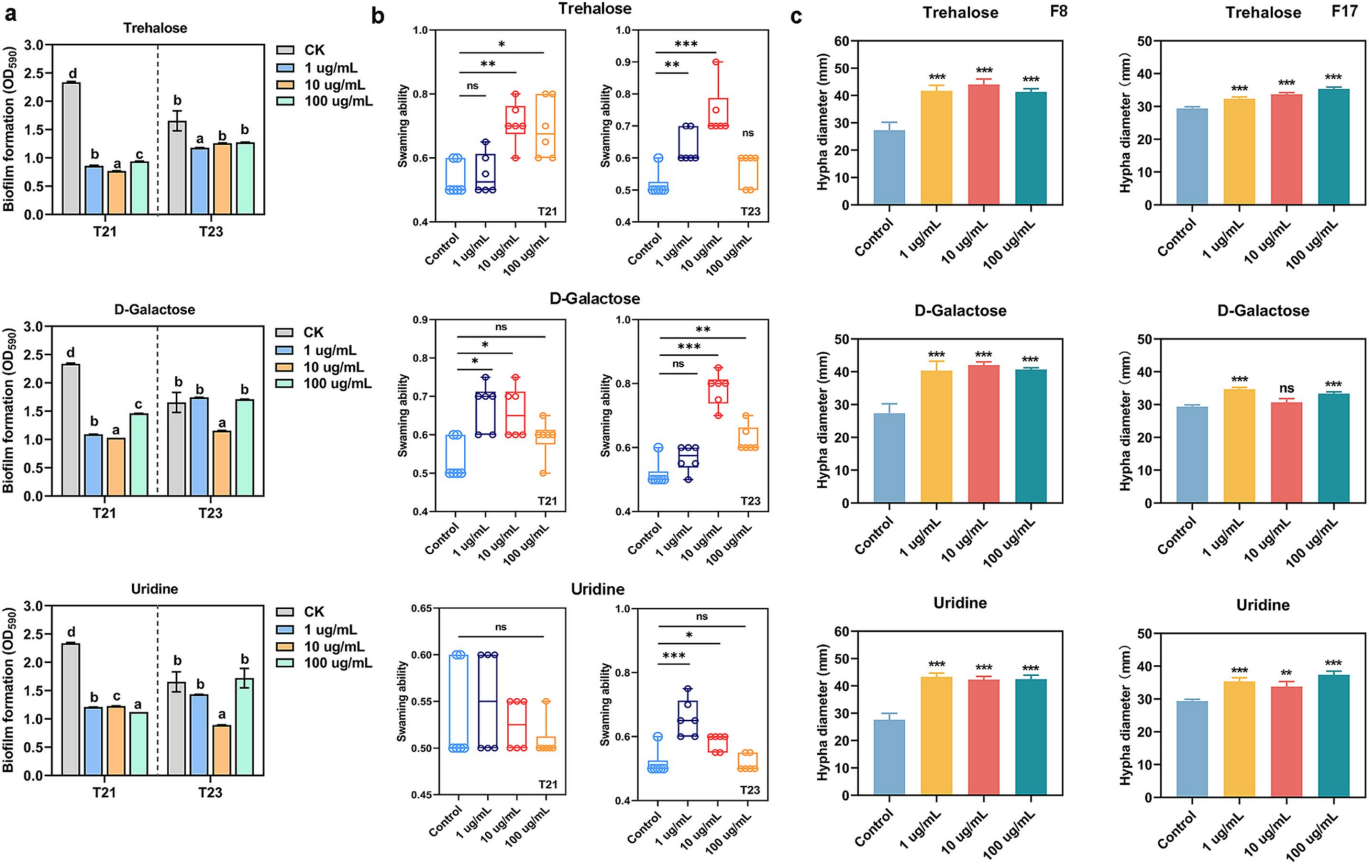
Effect of key compounds on antagonistic microorganisms and pathogens. **(a)** Biofilm formation. **(b)** Swimming ability. **(c)** Effect of key compounds on the growth of pathogen.

Further analysis of the effects of these metabolites on biofilm formation and swimming motility revealed that D-galactose, trehalose, uridine, and galactinol inhibited biofilm formation in both T21 and T23 at all concentrations tested (Figure 7). In contrast, 1 μg/mL and 10 μg/mL panose, as well as 10 μg/mL and 100 μg/mL xylobiose, enhanced biofilm formation in T23 (Figure 7a). Regarding bacterial motility, all compounds except uridine promoted the motility of T21 at specific concentrations. Notably, panose significantly enhanced the swimming motility of T21 at all tested concentrations. For T23, almost all the compounds significantly improved its swimming motility at a concentration of 10 μg/mL (Figure 7b).

These results indicated that the key metabolites can markedly influence the growth and key physiological traits of beneficial bacteria. However, the effects did not appear to be strictly concentration-dependent: the same metabolite could exert different effects on the same strain at different concentrations, and the regulatory effects also displayed distinct species-specificity between T21 and T23.

### 3.7 Trehalose significantly promotes the growth of pathogen

Based on the previous results, D-galactose, trehalose, and uridine exhibited consistently inhibitory effects on the beneficial antagonistic bacteria T21 and T23. Therefore, we further investigate whether these compounds enhance pathogen virulence by promoting pathogen growth (Figure 7c). The results showed that all three compounds significantly promoted the growth of both pathogens at nearly all tested concentrations, whereas they induced distinct alterations in the mycelial morphology of the pathogens. Specifically, although the mycelial growth area of F8 was significantly expanded, the hyphal structure appeared abnormally sparse and exhibited a typical ring-like growth pattern. Similarly, the hyphae of F17 grown in the presence of D-galactose and uridine were sparser than those in the control except under trehalose treatment, which might interfere with their pathogenicity. In contrast, trehalose significantly promoted pathogen growth without causing abnormal hyphal morphology, reflecting that it is more likely to exert a stable and direct physiological regulatory effect in the rhizosphere environment.

On the other hand, the KEGG analysis revealed that trehalose involved in multiple critical pathways, including plant secondary metabolite synthesis, starch and sucrose metabolism, suggesting that it may have a potential central regulatory role in responding to biological stress.

Based on the above considerations, trehalose was selected for further analysis to elucidate its potential role in the development of root rot.

### 3.8 Trehalose weakened the disease-suppressive effect of antagonistic microorganisms

In previous experiments, we confirmed that strains T21 and T23 exhibited significant disease control efficacy against root rot, while trehalose accumulated in the rhizosphere was found to promote pathogen growth. We therefore investigated whether trehalose modulates the disease-control and growth-promoting effects of T21 and T23. The results showed that trehalose application alone moderately reduced root rot incidence. However, when co-applied with antagonistic bacteria failed to yield a synergistic disease control effect, instead the disease-suppressive activity of both T21 and T23 was weakened. In the presence of trehalose, the disease incidence in the T21 and T23 treatments increased from 43.35–47% to 58–58.85%, and the disease index rose from 30–38.13 to 49.38–50.13. Meanwhile, the fresh weight, plant height, and root length of tea seedlings decreased significantly, with root length showing the most pronounced inhibition, indicating that trehalose substantially impaired the growth-promoting and disease-suppressive capacities of the antagonistic bacteria. Notably, the disease control efficacy of the synthetic microbial community (CpSC) was almost completely abolished by trehalose: the originally lowest disease incidence increased to similar to that of the trehalose-only treatment (Cp), and the growth-promoting effect was also markedly diminished. These results suggest that rhizosphere accumulation of trehalose not only directly stimulates pathogen growth, but also compromises the biocontrol function of beneficial microorganisms to exacerbating root rot development.

## 4 Discussion

The balance of “plant-microbe-metabolite” interactions in the rhizosphere micro-ecosystem is the core for maintaining plant health, while the microecological imbalance caused by pathogen invasion is the key driving factor for the occurrence and development of diseases (Berendsen et al., 2018; Zhou et al., 2023a). Focusing on tea root rot, this study systematically elucidated the changes in the rhizosphere microbiome and metabolome under pathogen infection, clarified the bidirectional regulatory role of key metabolites (especially trehalose) by suppressing beneficial microorganisms and promoting pathogen growth, and revealed the critical role of root exudates in weakening the disease-resistant function of the rhizosphere microbiome and exacerbating the occurrence of soil-borne diseases. This study provides a new theoretical perspective and practical basis for the ecological regulation and control of tea root rot through rhizosphere microbiome management.

It should be noted that the pathogen identified in this study, *Paraconiothyrium cyclothyrioides* F8 and *Apiotrichum sporotrichoides* F17, are significantly different in taxonomic status from the major pathogens previously reported to cause tea root rot include *Armillaria mellea*, *Candida* spp., and *Fomes lamoensis*, *Fusarium* and *Aspergillus*. However, there are no explicit reports indicating that *Paraconiothyrium cyclothyrioides* and *Apiotrichum sporotrichoides* can cause tea root rot (Tang et al., 2024; Ye et al., 2025; Zhao et al., 2025). This may be attributed to significant differences in climatic conditions, soil physicochemical properties (e.g., pH, nutrient content), and cultivation management practices (e.g., fertilization, intercropping patterns) across tea gardens, which resulting in the diversity of tea root rot pathogens, and our results further support that the disease can be caused by co-infection with multiple pathogens (Gu et al., 2019; Shao et al., 2025; Zhang et al., 2025). In the present study, multi-infection significantly increased the incidence of root rot, suggesting that the impact of mixed infection should be fully considered in disease management strategies, and targeted synergistic control measures should be adopted accordingly.

Numerous studies have demonstrated that pathogen invasion-induced differential assembly and functional adaptation of the rhizosphere microbiome, which represents typical microecological characteristics associated with plant disease development (Carrión et al., 2019; Zhou et al., 2023b). Our research found that both the bacterial and fungal communities in the rhizosphere of tea plants were significantly reshaped following tea root rot infection, PCA analysis showed a clear separation between the microbial communities of diseased and healthy plants (Figures 2a–d). In contrast to reported in crops such as citrus and *Panax notoginseng*, where microbial community diversity and richness decreased upon pathogen infection (Li et al., 2022; Wang et al., 2024), our study showed significantly higher *α*-diversity in the r rhizosphere microorganisms than that of healthy plants, which is consistent with previous studies showing increased α-diversity in rice leaves infected by pathogens such as *Magnaporthe oryzae* and *Xanthomonas oryzae* (Xu et al., 2025; Yang et al., 2020). In the early stages of pathogen infection, plant roots may actively recruit beneficial or antagonistic microorganisms by secreting specific metabolites to enhance defense, leading to a temporary increase in microbial species richness, which is known as the cry-for-help hypothesis. Although the enrichment of protective rhizosphere microorganisms in plants under pathogen pressure has been repeatedly validated, not all community shifts are adaptive or host-oriented (Koeberl et al., 2013; Liu et al., 2024). On the contrary, when this recruitment fails to effectively suppress the pathogen, it may lead to the proliferation of opportunistic microorganisms, resulting in a disruption of community homeostasis (Thomazella et al., 2025). Our results revealed a significant decrease in the abundance of *Basidiomycota* and a marked enrichment of pathogen fungi such as *Fusarium* and *Apiotrichum* in diseased plants rhizosphere. Concurrently, the abundance of beneficial fungi like *Saitozyma* and *Trichoderma*, as well as beneficial bacteria such as *Bacillus* and *Sporosarcina*, was significantly reduced (Figures 2e,f; Supplementary Figures S2, S3). This “trade-off” pattern indicates a transition of the tea rhizosphere microbiome from a healthy toward diseased state, suggesting that its stability and resistance to pathogen invasion may have been severely compromised.

As the core signaling molecules regulating the assembly of the rhizosphere microbial community, root metabolites have been extensively demonstrated to play a significant role in influencing the colonization and competition of rhizosphere microbiome (Kawasaki et al., 2021; Ma et al., 2022). This study revealed that pathogen infection significantly reshaped the root metabolic network, lipids and lipid-like molecules as well as phenylpropanoids accounted for the highest proportions among the 498 identified differentially metabolites identified (Figure 4). When confronted with pathogen attack, plants typically activate multiple defense responses, including cell wall reinforcement, reactive oxygen species (ROS) burst, and synthesis of secondary metabolites, which are involved in plant stress resistance and microbe-plant interactions (Chandrasekaran et al., 2025; Liu et al., 2023). For instance, phenylpropanoid compounds serve as key precursors for plant cell wall components like lignin and phytoalexins, the activation of these metabolic pathways and variations in their contents may strengthen the physical defense barrier of plant roots and restricting pathogen spread (Blaschek et al., 2023). Lipid molecules, meanwhile, function as signaling molecules mediating recognition interactions between plants and microbes such as mycorrhizal fungi (Jiang et al., 2017).

It should be noted that the levels of compounds associated with stress resistance and growth regulation, such as abscisic acid (ABA), gibberellins (GAs), and quercetin, were significantly higher in healthy plants rhizosphere compared to diseased plants (Supplementary Figure S3). The high-level accumulation of these compounds not only endows the healthy tea plants with direct pathogen-suppressive capacity, but also further enhances plant disease resistance by regulating microbial community structure. Flavonoids such as quercetin have been extensively documented to selectively recruit beneficial microorganisms like *Trichoderma* and *Bacillus* for rhizosphere colonization while suppressing certain pathogens (Wang et al., 2024; Yan et al., 2025). Similarly, we observed higher abundances of beneficial genera *Bacillus* and *Trichoderma* in healthy rhizosphere. Additionally, ABA and GAs may also function as microbial-recognizable signals that influence the tropism and colonization of specific microbial groups to maintain rhizosphere microbial homeostasis (Lopes et al., 2023). In contrast, the decreased of stress-related substances after pathogen infection may disrupt this synergistic defense network, lead to diminished intrinsic disease resistance in the tea plant and weakened recruitment signals for beneficial microbes, finally promoting disease progression.

Our research demonstrated that accumulated metabolites such as trehalose exhibited a significant positive correlation with pathogen like *Apiotrichum* and *Fusarium*, while exhibiting negative correlation with beneficial microbial genera such as *Sporosarcina*, *Bacillus*, and *Trichoderma* (Figure 5). These results suggested that pathogen infection-induced alterations in rhizosphere metabolites may shape an environment conducive to pathogen colonization by selectively enriching pathogen and suppressing beneficial ones, thereby exacerbating rhizosphere dysbiosis and promoting disease development. Among these metabolites, trehalose exhibited a particularly pronounced bidirectional regulatory effect. Here, we emphasize that due to the strong buffering capacity and inherent complexity of the soil environment, it is challenging to accurately determine the actual concentrations of tea root exudates. Therefore, we designed a gradient experiment (0, 1, 10, and 100 mg/L) to approximate a range of possible concentrations (cover the low, medium and high metabolite concentration) in the root environment. On the one hand, *in vitro* experiments confirmed that trehalose consistently inhibited the growth of beneficial antagonistic microbes T21 and T23. Specifically, trehalose significantly suppressed biofilm formation in these antagonistic bacteria. Given that biofilms serve as the foundation for microbial colonization, niche occupation, and functional expression in the rhizosphere, impaired biofilm formation implies reduced adsorption and colonization efficiency of antagonistic bacteria on tea root surfaces (Shi et al., 2024). Although trehalose enhanced the motility of some strains to a certain extent, it overall markedly weakened the competitive ability and colonization stability of the antagonistic bacteria in the rhizosphere (Figures 6, 7). On the other hand, trehalose significantly promoted the growth of both root rot pathogens (F8 and F17) *in vitro*, but induced specific morphological alterations in the pathogen mycelia, we speculate that that this may be attributed to differences in the metabolic utilization efficiency of trehalose among the pathogens (Figure 7). Specifically, F17 may possess a more efficient trehalose transport and metabolic system to sustain its growth and infection ability.

**Figure 8:**
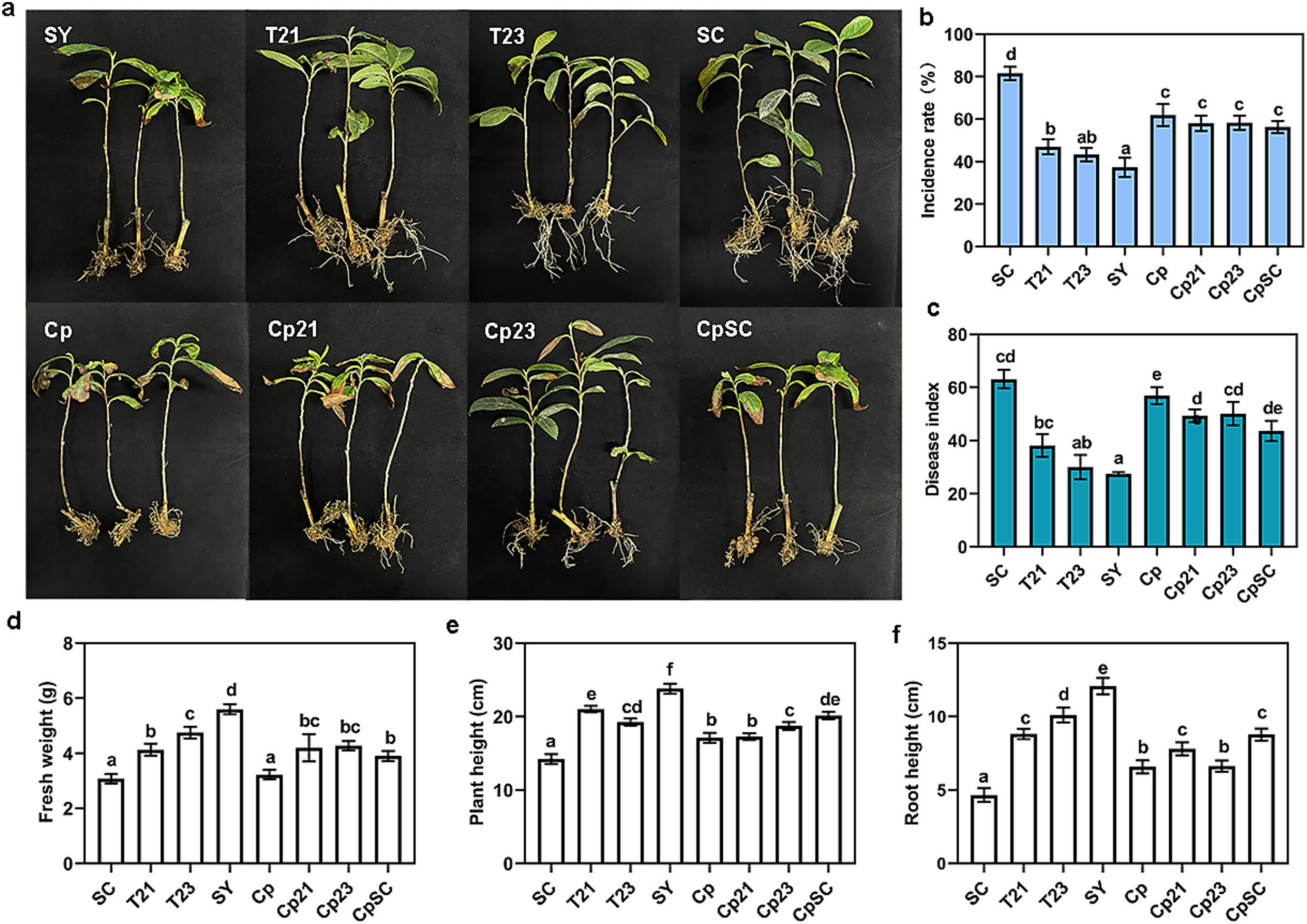
The effect of trehalose on the control of root rot disease by antagonistic microorganisms. **(a–c)** The incidence rate and disease index of different treatment tea plants. **(d)** Fresh weight. **(e)** Plant height. **(f)** Root length of different treatment tea plants.

It is noted that trehalose still moderately inhibited disease progression in pot experiments (Figure 8), and we speculated that exogenous trehalose may inhibit pathogen infection by inducing plant defense responses or regulate rhizosphere microbial community via promoting other beneficial microorganisms (Bi et al., 2025). Interestingly, pot experiments revealed trehalose play a double-edged sword role in the rhizosphere microenvironment: it not only nourished pathogens, but also significantly impaired the biocontrol efficacy of beneficial antagonistic strains *Sporosarcina pasteurii* T21 and *Lysinibacillus* sp. T23. This might be due to the fact that the pathogen has a more efficient trehalose metabolism ability, which enables the pathogen to preferentially utilize trehalose and fails to provide sufficient nutrition for the beneficial bacteria, thereby weakening the competitive advantage of the beneficial bacteria in the rhizosphere. Consequently, in the natural rhizosphere environment, the accumulation of trehalose following pathogen infection exerts a dual regulatory effect that simultaneously benefits the enemies (promoting pathogens) and impairs the allies (inhibiting antagonistic bacteria), which partially counteracts the efficacy of the biocontrol agents and ultimately leading to a marked increase in disease incidence. This also reveals the functional complexity of trehalose in the rhizosphere environment. It may have a direct nutritional effect on beneficial microorganisms or pathogens, while also acting as a signaling molecule to induce systemic disease resistance in plants or alter microbial interactions. In fact, such dual roles represent a common characteristic of many rhizosphere metabolites. Furthermore, validating the ecological and pathological significance of trehalose in the tea plant-pathogen-microbiome interactions at physiologically relevant concentrations represents a critical focus for our follow-up research.

Unlike common biocontrol agents such as *Bacillus* and *Pseudomonas*, the strains *Sporosarcina pasteurii* T21 and *Lysinibacillus* sp. T23 screened in this study exhibited excellent antagonistic activity (Figure 3). Notably, their combined application showed the most effective control of root rot, providing valuable microbial resources for the biocontrol of tea root rot. In particular, the combined application showed the optimal control efficacy against root rot, providing valuable microbial resources for the biocontrol of tea root rot. However, our study confirmed that trehalose impairs the disease-suppressive efficacy (Figure 8). Although the experiments were conducted under controlled conditions, this finding imply that the rhizosphere chemical environment is a critical determinant of biocontrol agents, while field conditions involve greater soil heterogeneity and more complex rhizosphere metabolite compositions. Plant-associated microbial communities are not simple reflection of surrounding microbial presence, instead, they are shaped by multifaceted interactions among the plant, microorganisms, and environmental factors (Cordovez et al., 2019; Trivedi et al., 2021). Studies have demonstrated that root exudates accumulated under continuous cropping can inhibit the abundance of beneficial rhizosphere microorganisms and release ecological niches that increase the invasion probability of soil-borne pathogens (Cao et al., 2025; Upadhyay et al., 2022). Therefore, enhancing the adaptability of beneficial microbial metabolites or modulating the rhizosphere chemical environment to maintain stable biocontrol efficacy may represent key directions for unlocking microbial community potential in sustainable agriculture.

**Figure 9:**
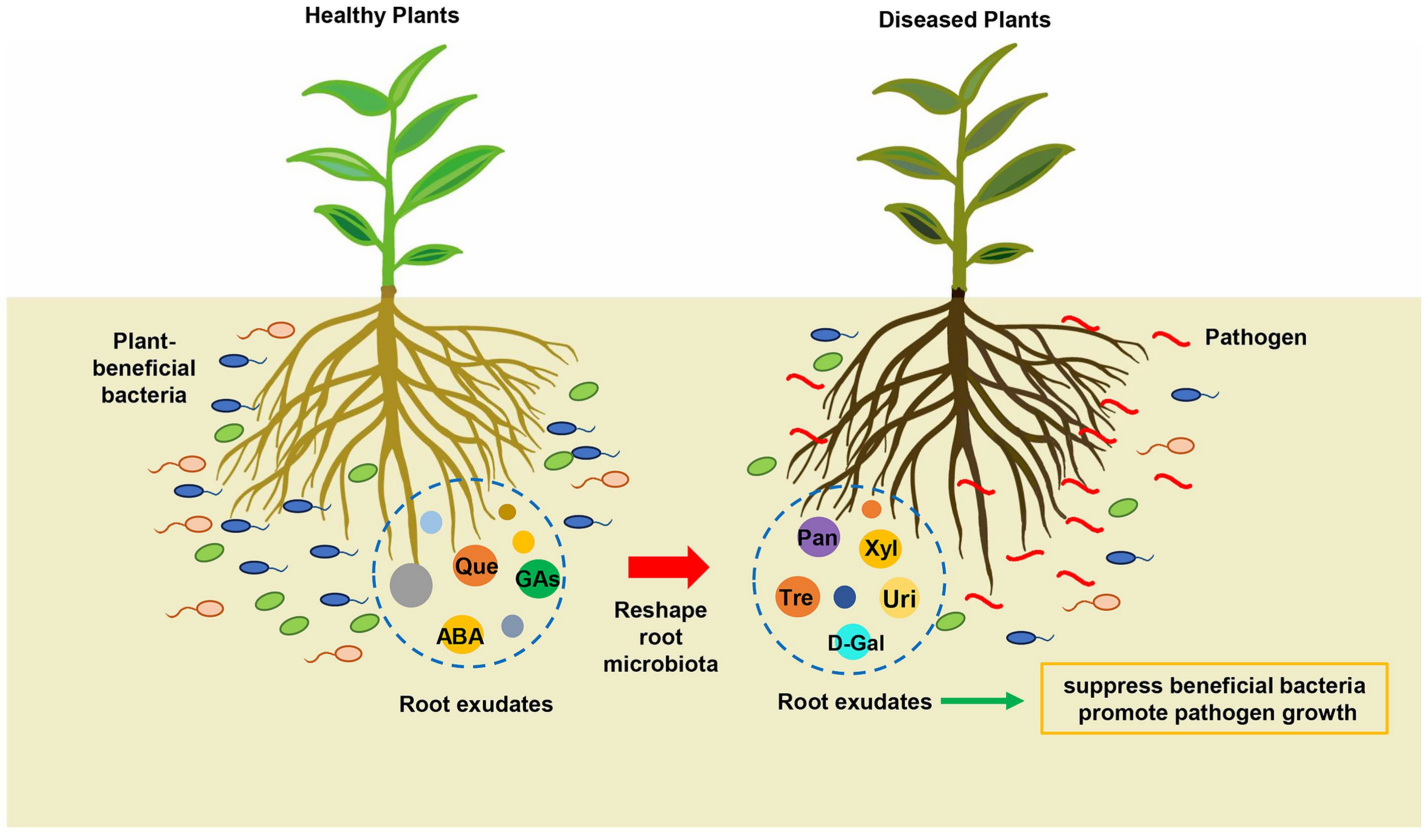
Schematic model of root exudate-mediated differences in rhizosphere microbial community assembly in diseased and healthy plants.

Thus, we propose the following hypothetical model: initial pathogen infection alters the root exudates of tea, resulting in the accumulation of specific metabolites represented by trehalose in the rhizosphere. These metabolites further promote pathogen growth and inhibit beneficial microorganisms, driving the rhizosphere microbial community toward a dysbiotic state that favors pathogen proliferation and virulence. Concurrently, the decline in stress-resistance related compounds like quercetin weakens the plant’s intrinsic immune capacity and its ability to recruit beneficial partners, ultimately exacerbating disease development and plant health deterioration (Figure 9). However, the molecular mechanisms underlying trehalose-mediated regulation of the interactions between beneficial microorganisms and pathogens remain to be further investigation.

## 5 Conclusion

Root rot disease is one of the most destructive and intractable diseases in tea cultivation, while the pathogenic mechanism remains unclear. In this study, we integrated microbiome analysis, metabolomics, and functional validation to systematically elucidate the microecological mechanisms of tea root rot. Our results demonstrated that the relative abundance of *Basidiomycota* was significantly reduced in the rhizosphere of diseased tea plants, while pathogen-associated genera such as *Fusarium* and *Apiotrichum* were significantly enriched. Meanwhile, the abundances of beneficial fungi such as *Saitozyma* and *Trichoderma*, as well as beneficial bacteria including *Bacillus* and *Sporosarcina* were marked decreased. Metabolomic analysis revealed the contents of D-Galactose, Uridine, Xylobiose, Panose, Glucomannan, Galactinol, and Amylopectin were significantly higher in the rhizosphere of diseased plants. Further research confirmed that trehalose played a bidirectional regulatory role: it suppressed biofilm formation and motility of the beneficial strains T21 and T23, while promoting the growth of the pathogens. This study clarified the critical role of trehalose in disease progression, emphasizing the dominant role of plant metabolites in promoting pathogen growth, inhibiting beneficial microorganisms, and driving the assembly of rhizosphere microbial communities from disease-suppressive to disease-susceptible states. Meanwhile, two rhizosphere beneficial bacteria (T21 and T23) with high antagonistic and growth-promoting activities were isolated and identified. Overall, this study enriches the theoretical system of microecological regulation of plant soil-borne diseases and provides important theoretical and practical basis for the green prevention and control of tea root rot.

## Data Availability

The datasets presented in this study can be found in online repositories. The names of the repository/repositories and accession number(s) can be found below: https://www.ncbi.nlm.nih.gov/, PRJNA1396795.
