# Supplementary material for: Trehalose-mediated reshaping of the rhizosphere microbiome drives tea root rot progression
Source: Front Microbiol. 2026 Feb 19;17:1787317. doi: 10.3389/fmicb.2026.1787317 (PMC12961617; doi:10.3389/fmicb.2026.1787317)
Supplement: Supplementary file 1 [file Supplementary_file_1.docx]

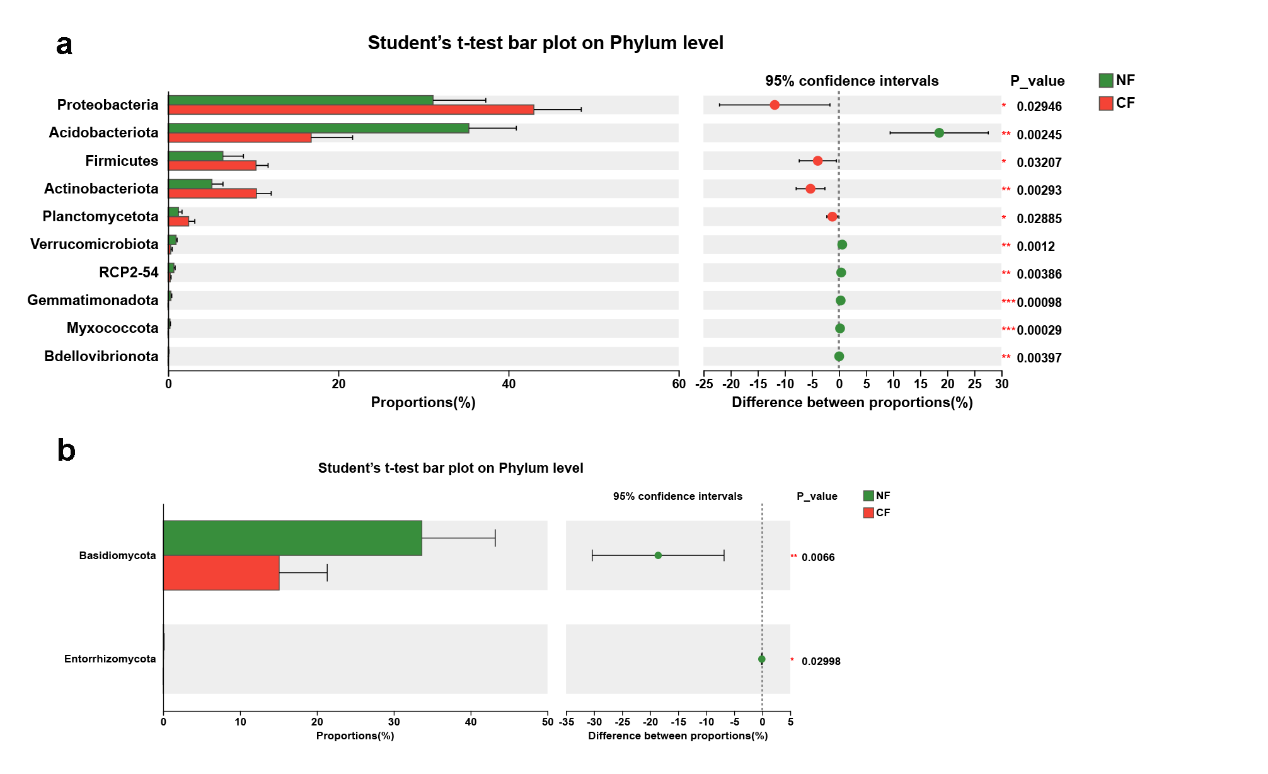
**Figure S1 Abundance analysis of bacteria (a) and fungi (b) at the phylum level in the rhizosphere of healthy and diseased tea plants.**


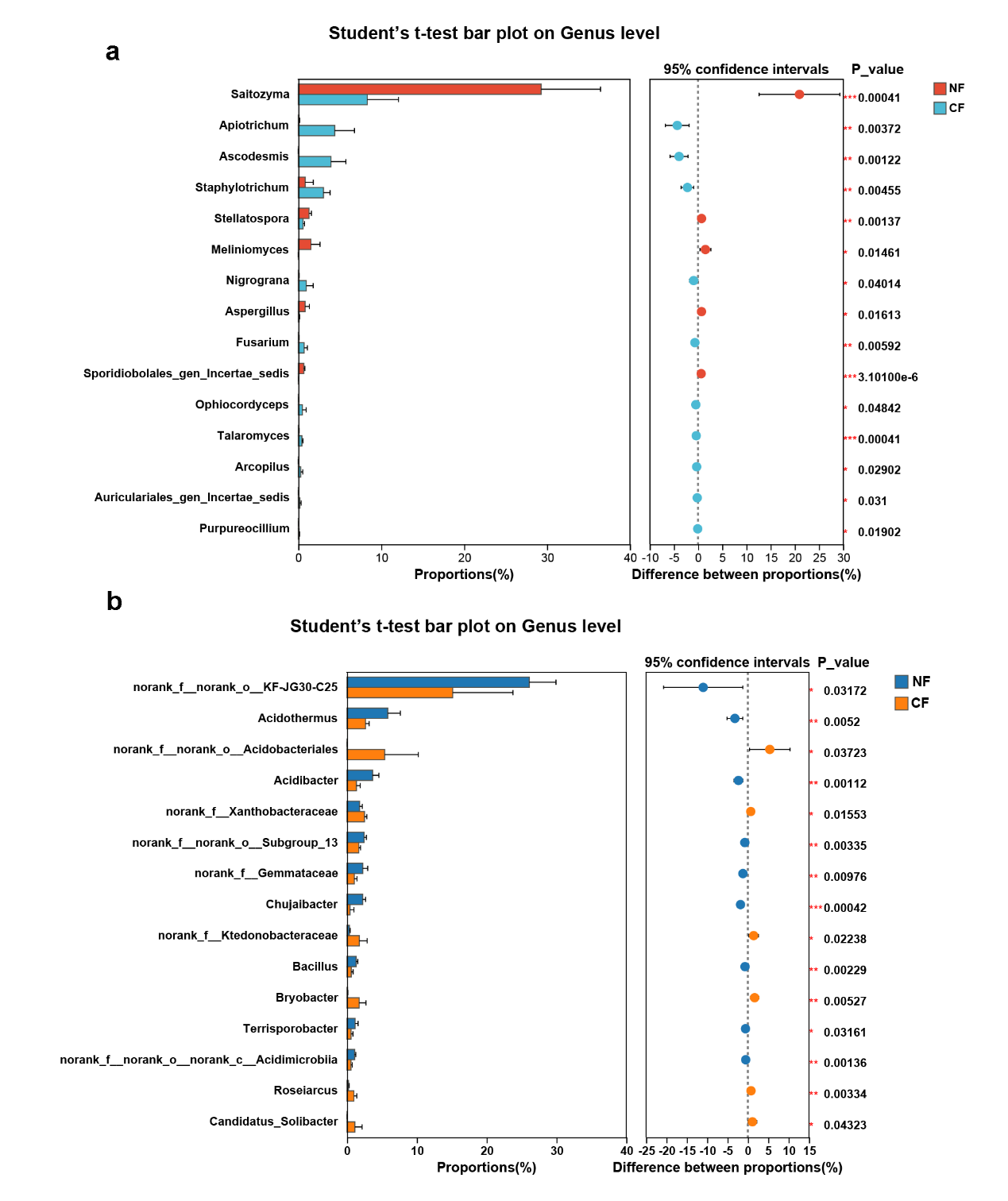
**Figure S2 Abundance analysis of fungi (a) and bacteria (b) at the genus level in the rhizosphere of healthy and diseased tea plants.**


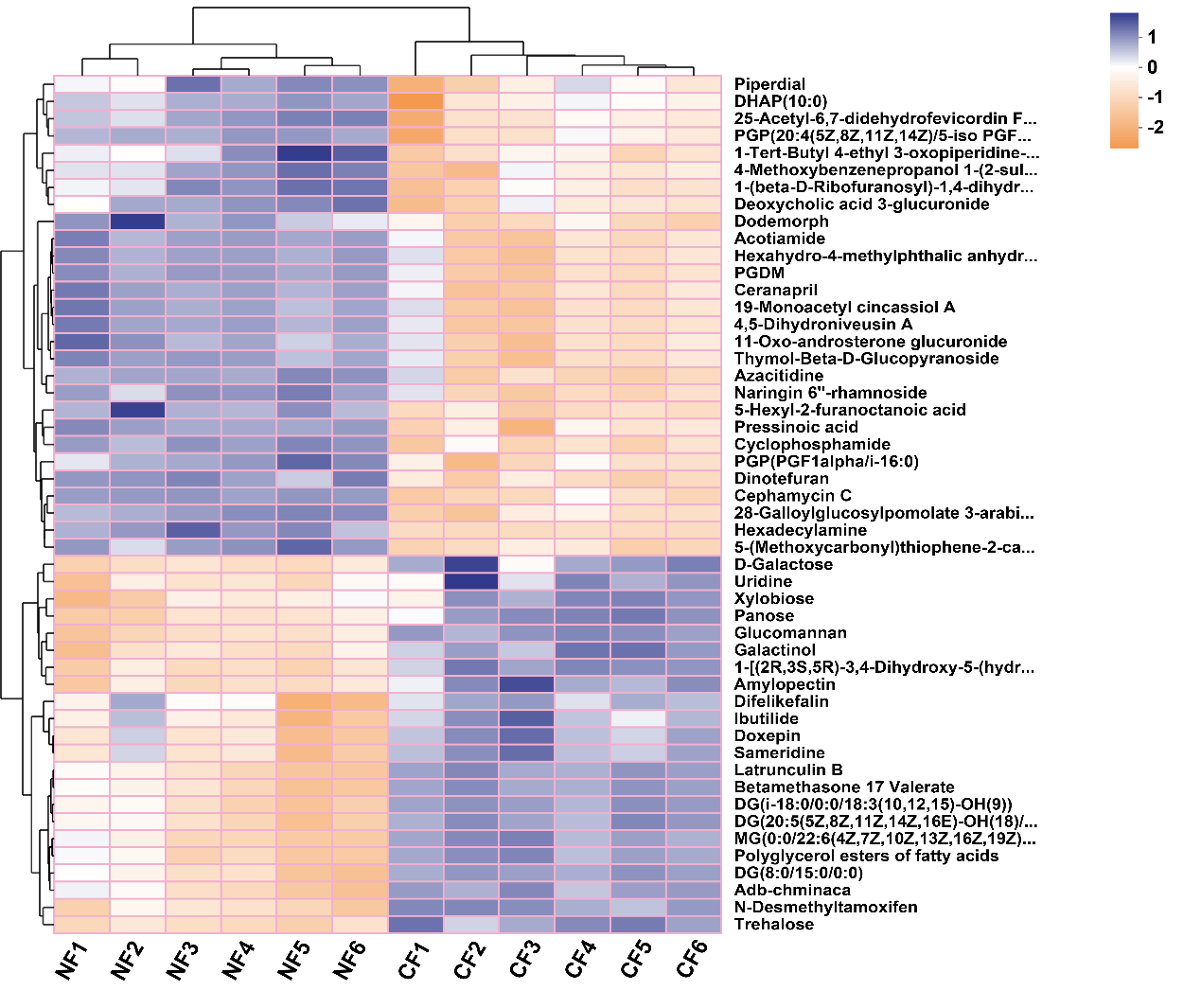
**Figure S3 Heat map analysis of the top 35 differential metabolites in the rhizosphere of healthy and diseased tea plants.**


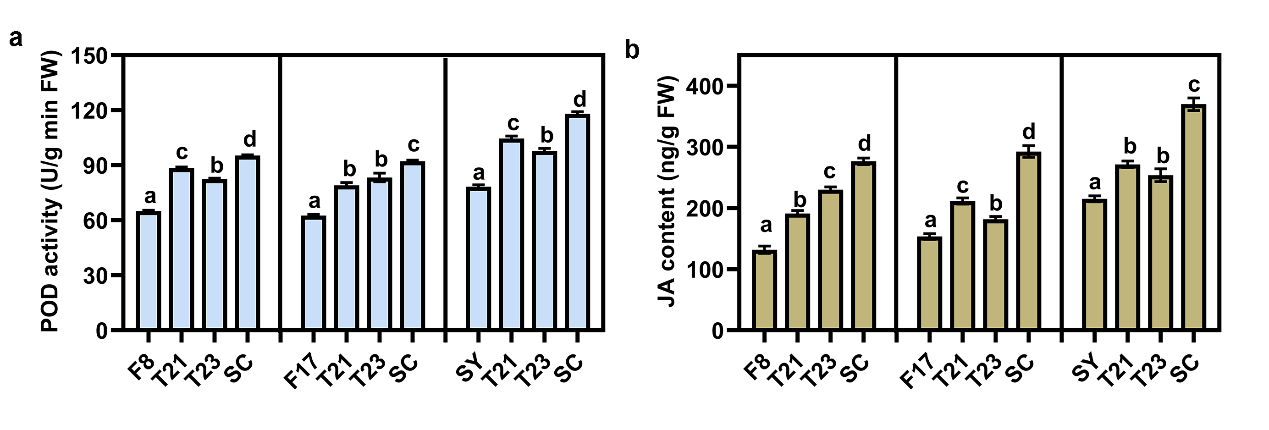
**Figure S4 The POD enzyme activity (a) and the content of JA (b) in tea roots with antagonistic microorganisms control root rot disease**


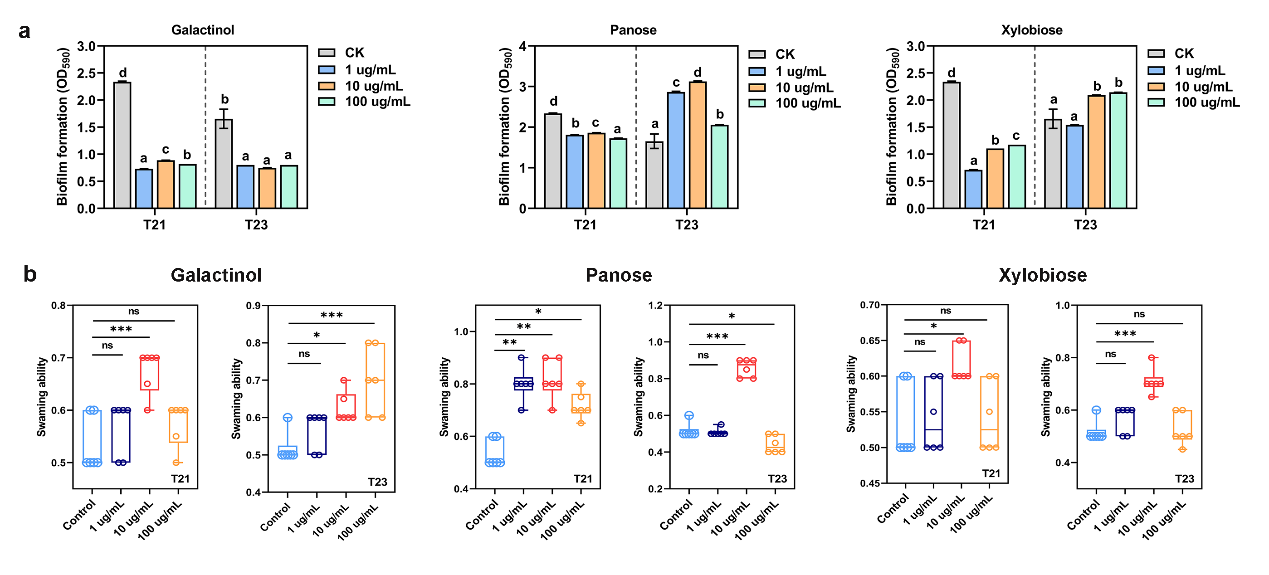
**Figure S5 Effect of key compounds on antagonistic microorganisms and pathogens.** (a) Biofilm formation. (b) Swamming ability.


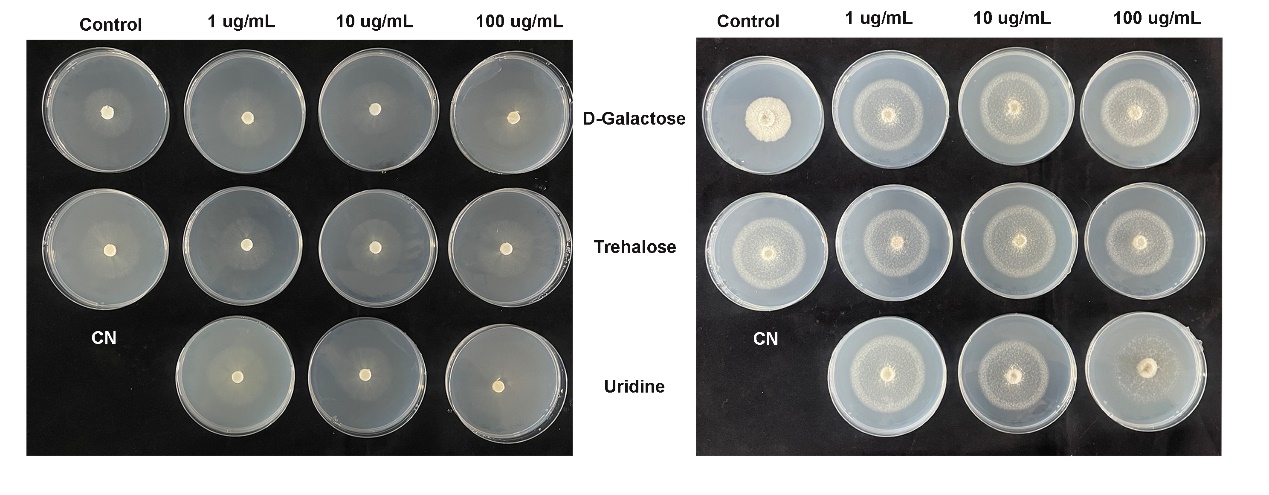
**Figure S6 Effect of key compounds on the morphology of pathogen hyphae.**


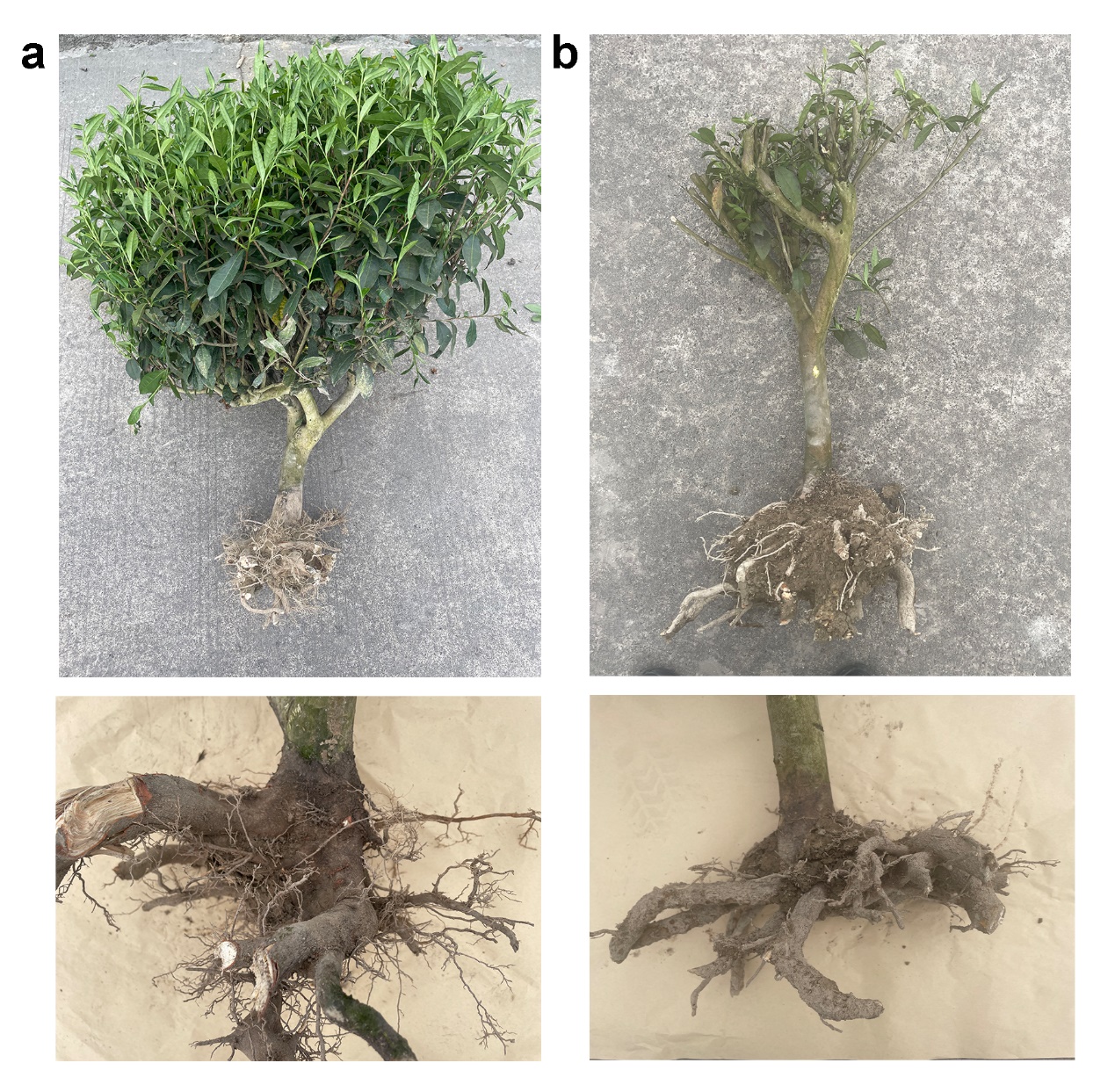
**Figure S7 Healthy tea plant (a) and diseased tea plants (b) in the fields.**
